# Guernsey’s Bœnninghausen

**Published:** 1888-02

**Authors:** 


Guernsey's Bœnninghausen.

Dr. William Jefferson Guernsey has issned a pro-pectus of a new edition of Bœnninghausen's famous *Therapeutic Pocket-book.* His idea is: "To reproduce the entire work, not in book form, but on slips of paper, each slip bearing the names of one hundred and twenty six remedies given in the book, and opposite those homœopathic to the symptom numerals to designate their value, corresponding to the grade of medicines used in the book. For instance: the figure 4 will indicate LARGE CAPS; 3, SMALL CAPS; 2, *Italics;* and 1, Roman. Remedies that are not recorded with the symptom will have a blank space opposite. Take, for example, the symptom of 'Longing for fruit.' Under this head, Bœnninghausen gives eight remedies—*Alum.*, *Ant. tart.*, Chin.,

IGNAT., Magn. cb., Puls., SUL. AC., VERAT. On the paper representing this symptom will appear the entire list of the one hundred and twenty-six remedies, and opposite *these eight remedies* will be placed numerals to designate their value, as follows: Alum, 2; Ant. tart., 2; Chin., 1; Ignat., 3; Mag. cb., 1; Puls., 1; Sul. ac., 3; Verat., 4, while all the *other medicines* will be *unnumbered*. Now, let us suppose a case to present seven symptoms. These symptoms are hunted out in the index or key, and the seven corresponding papers placed side by side on the table, when a glance across the sheets will show what medicines are given on *all* the papers, and *these alone* are noted. *An addition of the numbers opposite each of these remedies will show which ranks greatest in importance.* Should two or more sum up the same amount the Materia Medica must be consulted to ascertain which is the more closely allied to the case.

"It is not expected that this plan can be used with every prescription, but is intended for those cases for which it is particularly difficult to find the remedy."

